# Editorial: Highlights in nano-based drug delivery 2021/22

**DOI:** 10.3389/fmedt.2023.1130414

**Published:** 2023-02-24

**Authors:** Santosh Kumar, Helena F. Florindo, Gianfranco Pasut

**Affiliations:** ^1^ Department of Chemistry, School of Basic & Applied Sciences, Harcourt Butler Technical University, Kanpur, India; ^2^ Research Institute for Medicines (IMed.ULisboa), Faculty of Pharmacy, Universidade de Lisboa, Lisbon, Portugal; ^3^ Pharmaceutical and Pharmacological Sciences Department, University of Padova, Padova, Italy

**Keywords:** 3D bioprinting, hydrogels, brain delivery, gene therapy, multiple sclerosis

**Editorial on the Research Topic** Highlights in nano-based drug delivery 2021/22

We are pleased to introduce the Research Topic *Frontiers in Medical Technology – Highlights in Nano-Based Drug Delivery 2021/22*. The articles published in this brief collection provide an important contribution to the field of nano-based delivery.

The paper *Modeling the Three-Dimensional Bioprinting Process of β-Sheet Self-Assembling Peptide Hydrogel Scaffolds*, authored by Chiesa and colleagues, describes an interesting approach of extrusion-based three-dimensional (3D) bioprinting. 3D bioprinting is fast changing the field of personalized healthcare offering new approaches and processes for the fabrication of scaffolds, living tissues, unique drug dosage forms, etc. This paper highlights the limitations of the common trial and error approaches, which are frequently used for achieving the desired final features of the designed structures. To address such issue, the authors investigated the potential of finite element simulation in predicting the printability of a biomaterial. Thanks to this study, the authors successfully 3D bioprinted human ear-shaped scaffolds by using self-assembling peptide hydrogels with a poly(vinyl alcohol)–poly(vinyl pyrrolidone) copolymer. This methodology can have a broad applicability, thus further promoting the expansion of 3D bioprinting.

Gene therapy has finally climbed the mountain of efforts and has become a real therapy option, with enormous future applications and great expectations. Gene therapy has grown thanks to improvements on gene delivery that takes care of the proper transportation of the genetic material to the target cells *via* viral or non-viral carriers. Pardridge's review *Brain Delivery of Nanomedicines: Trojan Horse Liposomes for Plasmid DNA Gene Therapy of the Brain* offers a great overview of the requirements needed for the development of an ambitious project on brain gene therapy. The challenge, as it can be easily understood, is huge owing to the intrinsic difficulties encountered in crossing the blood-brain barrier. Here, you can find a precise analysis of all relevant components that can guide you towards the successful delivery of genetic material to the brain.

Another relevant investigation has been conducted by de la Flor and colleagues. In their paper *Multiple Sclerosis: LIFNano-CD4 for Trojan Horse Delivery of the Neuro-Protective Biologic “LIF” Into the Brain: Preclinical Proof of Concept*, the authors studied the potential of a nanoformulation of the cell growth factor LIF for the treatment of multiple sclerosis. Since such disease still represents an unmet medical need and this study can advance the development of new therapeutic approaches, in which thanks to a nanomedicine properly designed, the bioactive molecule is delivered precisely at the site of action.

We hope that you will enjoy the papers contained in this Research Topic, which we hope will inspire your future scientific endeavors.

